# The Healing Effect of N-Hexan- Dichloromethane Extract Root *Onosma Bulbotrichum* in Second Degree Burns

**Published:** 2018-01

**Authors:** Aliasghar Hemmati, Forough Namjuyan, Sadegh Yousefi, Gholamreza Housmand, Hossein Khadem Haghighian, Anahita Rezaei

**Affiliations:** 1 Department of Pharmacology Toxicology, School of Pharmacy, Physiology Research Center, Jundishapur University of Medical Sciences, Ahvaz, Iran;; 2 Department of Pharmacognosy, School of Pharmacy, Ahvaz Jundishapur University of Medical Sciences, Ahvaz, Iran;; 3 Department of Pharmacology, School of Pharmacy, Ahvaz Jundishapur University of Medical Sciences, Ahvaz, Iran;; 4 Department of Nutrition, Faculty of Health, Qazvin University of Medical Sciences, Qazvin, Iran;; 5 Department of Pathology, Faculty of Veterinary Medicine, Shahid Chamran University, Ahvaz, Iran

**Keywords:** Burn, Healing, Silver sulfadiazine, *Onosma bulbotrichum*, Rabbit

## Abstract

**BACKGROUND:**

Wound healing is the process of repair following an injury to the skin and other soft tissues. In this study, the effect of n-hexane d-chloromethane extract (1: 1) of root *Onosma bulbotrichum* DC on the second degree burn in rabbit model was investigated.

**METHODS:**

Thirty-six adult rabbits of both sexes were randomly divided into six groups, control (without treatment), negative control (treatment with cold cream), positive control (treatment with silver sulfadiazine), and treatment groups with 5%, 1% and 2% *O. bulbotrichum* cream and assessed histologically.

**RESULTS:**

The best result was shown in 5% *O. bulbotrichum* group similar to silver sulfadiazine group. The maximum amount of collagen and the tensile strength of tissue were observed in 5% *O. bulbotrichum* and silver sulfadiazine groups. Histopathological examination showed that burn healing in treatment group with 5% *O. bulbotrichum* was faster than other groups.

**CONCLUSION:**

The 5% *O. bulbotrichum* cream was shown to have healing, and anti-inflammatory effects when used in treatment of second degree burns.

## INTRODUCTION

The final aim of burn management and therapy is wound healing and epithelization as soon as possible in order to prevent infection and to recover functional and aesthetic effects.^1^ The use of topical chemotherapy has been fundamental in this regard and has helped to improve the survival of patients with major burns and to minimize the incidence of burn wound sepsis, a leading cause of mortality and morbidity in burn patients.^2^ Perhaps the most traumatic injury to the victim, over the years has been burns treated with various wound coverings,^3^ both natural and synthetic aiming to find the most efficient means to control the damage that go beyond the site of the injury.^4^

It is important as well to promote healing, since the primary goal when it comes to the treatment of burns is the physiological closure in the shortest possible time.^5^ Among the complications associated with thermal injury, the formation of oxygen free radicals that can produce infection showed that 50% to 75% of mortalities in the hospital are due to an injury.^6^ Burn recovery is the process of spontaneous and without the need for other factors, but infection is needed to for care.^7 ^Wound healing has been widely discussed in the medical literature.^8^

In topical burn therapy, silver sulfadiazine was introduced as the gold standard having antibacterial properties too.^9^ Numerous studies were carried out to develop more sophisticated dressings to expedite healing process and diminish bacterial burden in wounds.^8^ Even medicinal plants were introduced in wound healing of burned injuries, traditional forms of medicine, especially herbal products, which have been employed for centuries in Africa and Asia are under scientific investigation for their roles in wound treatment.^10^^,^^11^ There are many reports confirming the use of medicinal plants for dressing of wounds who was described by Avicenna, the Persian physician (980-1037 AD) in his famous book, Canon of Medicine.^2^

The family Boraginaceae have about 100 genera and 2000 species that are often found in warm and temperate regions.^12^

Genus Onosma has over 150 species and mostly in the climate of hot and dry areas, especially in the Mediterranean and Asia and mainly in Iran.^13^ The most important compounds isolated from this genus, such as the naftoquinona, shikonin, and alkannin. In the root, a red pigment is present in different ratios.^14 ^For example, species such *Onosma argentatum* has 3-hydroxy-isovaleryl shikonin^5^ and O-dimethyl acetyl shikonin.^15^ These compounds are lipophilic and have several pharmacological activities, such as wound healing, antimicrobial, antifungal, anti-inflammatory, anticancer, and antioxidant activities and have topoisomerase inhibitors.^16^^,^^17^

The root is the most important part of this genus that is used for treatment of wounds, burns, and hemorrhoid.^18^ Previous study has shown that the methanol extract of the root *Onosma hispidum* is very effective for wound healing in animal models.^19^ In addition, extract prepared from the *Onosma stenosiphon* was found to improve burn in 24 days in rats.^20^ In the present study, we investigated the effect of protective n-hexane-dichloromethane extract (1:1) root *Onosma bulbotrichum* DC in second degree burns in a rabbit model.

## MATERIALS AND METHODS

The plant was collected in the West region of Iran and its root was separated, and then dried. Soxhlet method was used for preparation of n-hexane solvent dichloromethane (1:1) extract. Three hundred g of the dried root of the plant, were divided into 4 sections and each section (75 g) was transferred to a filter paper and then, 250 ml of n-hexane solvent dichloromethane (1:1) in each of the balloons were added, heated for 4 hours until the temperature reached 40°C, and finally filtered for extraction.^21^ In the next stage, the extract was concentrated by rotary and further the concentrated extract of steam-bath was used as a solvent, evaporated completely and dried. The 5%, 1% and 2% of the extract was prepared as cold cream.

Healthy male and female rabbits (1.8 to 1.2 kg) were obtained from laboratory animal care center of our institution and kept in the animal room of School of Pharmacy with 12 hours light, 12 hours dark and 22±2°C temperature maintenance and of intensive food, carrots, lettuce and water used without limitation. These animals were placed in the standard cages. The burn area was created on the back of the animals, near the spine while, the area was completely shaved and disinfected with ethanol. For local anesthesia 2% lidocaine was used. To induce burn, a circular steel as diameter of 2.5 cm was heated up to 150°C and was located on the animal’s skin for 20 seconds to create the burn. Three times in day, the outline of each wound was traced on a transparent plastic sheet. 

Then surface area of wounds was calculated. Area of the first day was 100 and the degree of healing was assessed every day compared with the first day.^22^ Groups were (i) Without treatment that did not receive any treatment, (ii) Negative control that received treatment with cold cream twice daily till the wounds completely healed, (iii) Positive control that received treatment with silver sulfadiazine cream twice daily till the wounds completely healed, (iv) Test 1 that underwent treatment with 5% *O. bulbotrichum* cream twice daily until the wounds completely healed, (v) Test 2 that was treated with 1% *O. bulbotrichum* cream twice daily until the wounds completely healed, and (vi) Test 3 that had treatment with 2% *O. bulbotrichum* cream twice daily until the wounds completely healed.

After 7 and 14 days, skin sample was provided for histological study. Samples were fixed in 10% formalin and typical thickness of 5 micrometers were prepared from sections. In the next stage, the sections were stained with hematoxylin and eosin and visualized by light microscopy. Samples were also incubated with 10 ml of 0.1% Sirius Red and 0.1% Fast Green saturated with picric acid dissolved in water for 30 min, then was carefully decolorized and washed in deionized water until becoming colorless. Then, 1 ml of color solution was added to the tissue blocks and slowly shaken, and was transferred carefully in microtubes. Absorption was read in a wavelength of 540 and 605 nm using a spectrophotometer.^23^

The amount of collagen and non-collagen proteins was calculated using the following formula: Collagen (µg/section)=[OD540-(OD605×0.291)]/37.8×1000, non-collagen protein (µg/section)=OD605 values/2.04×1000, total protein=collagen+non-collagen protein. In the end of treatment period, skin with dimensions 20×5 mm from the wound was removed and the tensile strength was measured with tensiometer.^24^ All treated groups were compared with the control group and the results were analyzed statistically using one-way ANOVA and followed by Tukey test to identify the differences between treated groups and the control. The data were considered significant at *p*<0.05.

## RESULTS

**Fig. 1:**
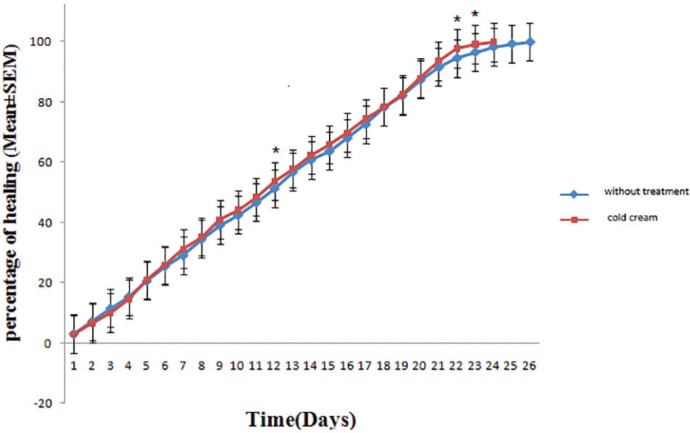
Comparison of burn healing after treatment with groups of cold cream and without treatment.

**Fig. 2:**
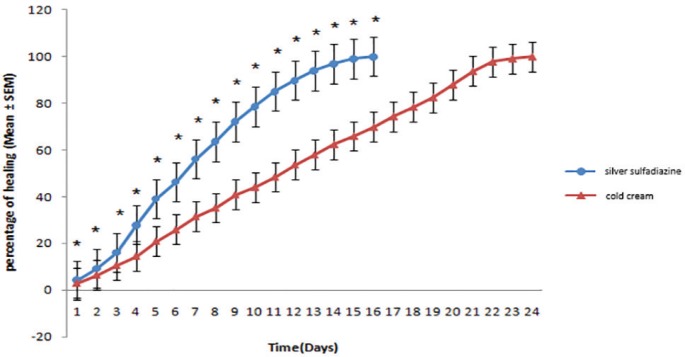
Comparison of skin wound healing after treatment with silver sulfadiazine and cold cream.

In the Group of rabbits left without treatment, healing lasted 26 days. In comparison to treatment group with cold cream (negative control), there was significant differences after 12, 22 and 23 days, but on other days, there was not any significant difference (Figure 1). In the group of treatment with silver sulfadiazine, burn healing lasted 16 days and in comparison to the negative control group and the group without treatment, a significant difference was noted for all days (Figure 2).

**Fig. 3:**
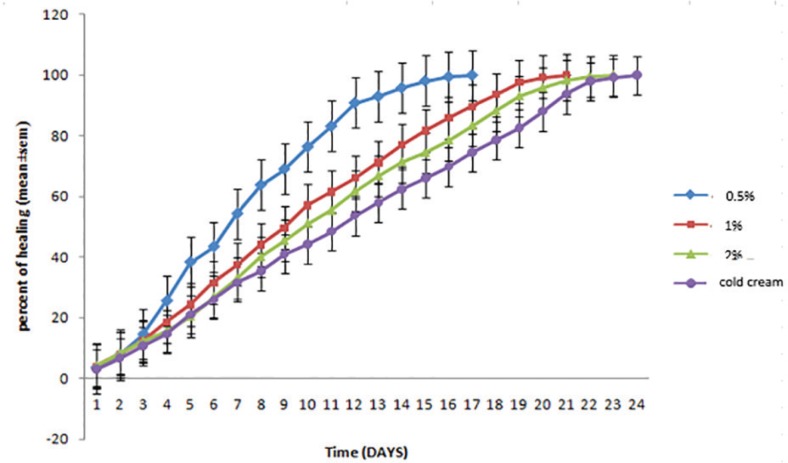
Comparison of the rate of healing of skin wounds in groups treated with different concentrations of *O. bulbotrichum *cream (5%, 2%, and 1%) compared the group treated with cold cream.

**Fig. 4:**
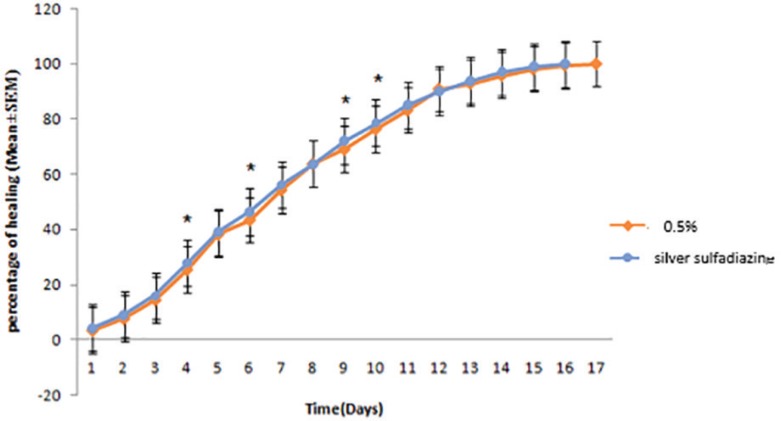
Comparison of skin wound healing after receiving 5% *O. bulbotrichum* cream and treatment group with silver sulfadiazine.

In the treatment group with cold cream (negative control), burn healing lasted 24 days and in comparison to treatment group with 5% *O. bulbotrichum* cream, a significant difference was seen for all days except for the first day. However, when compared to treatment group of 1% *O. bulbotrichum* cream, a significant difference was visible for all days. In treatment group with 2% *O. bulbotrichum* cream, a significant difference was observed for all days except for days 4, 5, and 6 (Figure 3). In the treatment group with 5% cream, burn healing was lasted 17 days. In compression between treatment groups with 5% *O. bulbotrichum* cream when compared to the silver sulfadiazine group, a significant difference was noticed for days 4, 6, 9 and 10 (Figure 4).

**Fig. 5:**
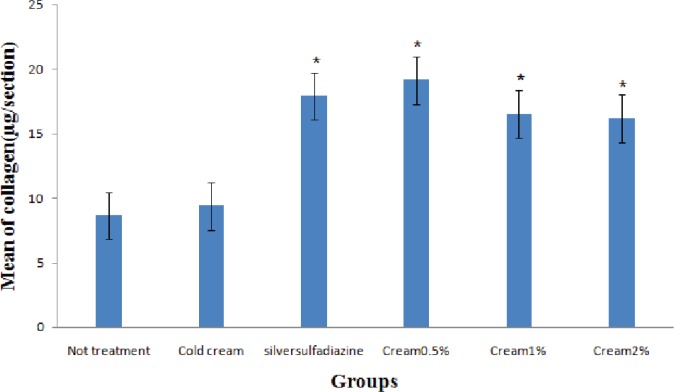
Comparison of collagen concentrations (µg/section) in all groups.

**Fig. 6:**
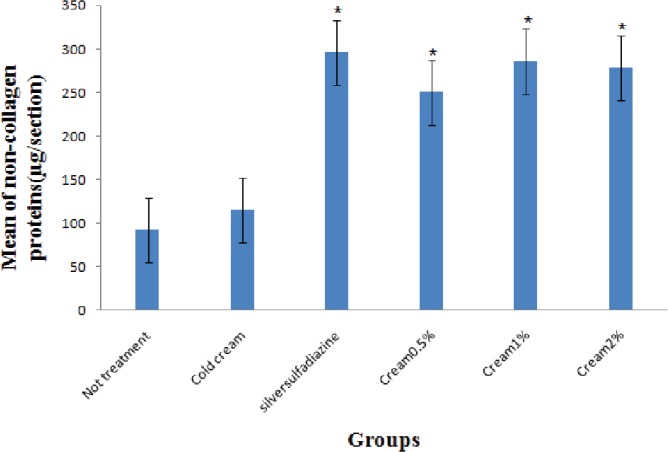
Comparison of non-collagen protein concentrations (µg/section) in all groups.

Also, the maximum amount of collagen was shown in the treatment groups with 5%, 1%, and 2% *O. bulbotrichum* cream and silver sulfadiazine and the minimum amount in without treatment group and the negative control (Figure 5). The highest rate of non-collagen proteins was observed in silver sulfadiazine treatment group when compared to other groups (Figure 6). The groups treated with 5% silver sulfadiazine and *O. bulbotrichum* cream, revealed the maximum amount of tissue resistance.

**Fig. 7:**
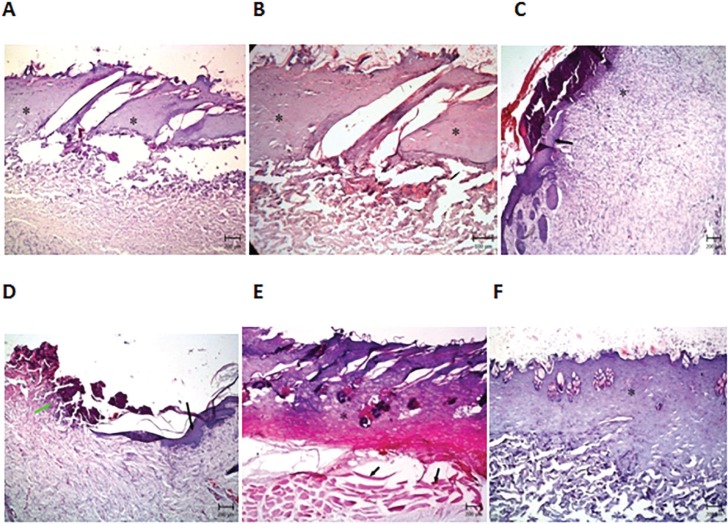
Comparison of histological changes in different groups during the first week: In the group without treatment (A), treatment with cold cream (B), positive silver sulfadiaine control (C) treatment group with 5% *O. bulbotrichum* cream (D), treatment group with 2% *O. bulbotrichum* cream (E) treatment group with 1% *O. bulbotrichum* cream (F).

**Fig. 8:**
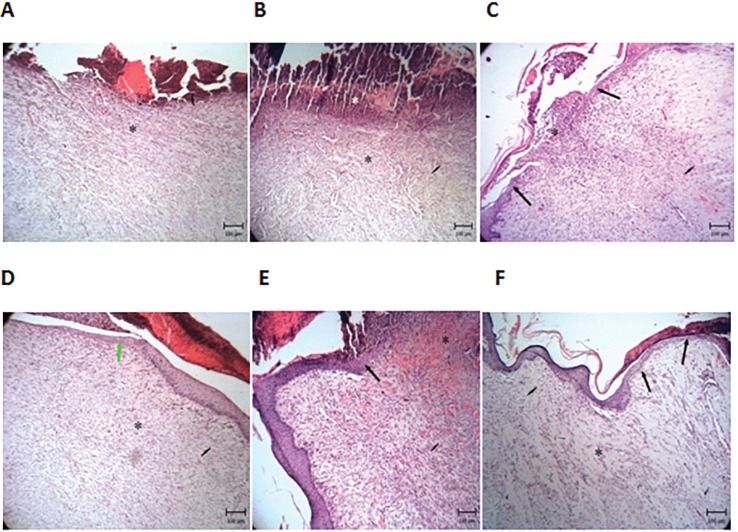
Comparison of histological changes in the second week: In the group without treatment (A), treatment with cold cream (B), positive silver sulfadiazine control (C) treatment group with 5% *O. bulbotrichum* cream (D), treatment group with cream 2% *O. bulbotrichum* (E) treatment group with cream 1% *O. bulbotrichum* (F).

Histological studies confirmed that in the group without treatment in the first week, necrosis and inflammation were visible (Figure 7A and 7B). In the second week, inflammation was continued and a few red blood cells were visible (Figure 8A and 8B). In positive control in the first week of treatment, inflammatory cells were shown (Figure 7C.), that after two week, proliferation and migration of cells were started to the burn region (Figure 8C). In treatment group with 5% *O. bulbotrichum* cream, in the first week; proliferation of epithelial cells was seen (Figure 7D). In the second week, proliferation was continued and observed as widely fleshy bud tissues that covered large regions of the burn injury area (Figure 8D). 

In the treatment group with cream 2% *O. bulbotrichum* in the first week, inflammation and presence of basophils were shown in the damage region (Figure 7E). In second week, there was inflammation, but epithelium cells increased and red blood cells were visible (Figure 8E). In the treatment group with 1% *O. bulbotrichum* cream, in the dermis, hypodermis, necrosis was observed in the first week (Figure 7F). Proliferation and presence of epithelial cells were shown in the second week (Figure 8F).

## DISCUSSION

Burns injuries are considered as the third major cause of accidental death in the world, in all age groups, being the most usual cause of scalding, and the home and the environmental greatest frequency of accidents, making up 60% of total events.^25 ^Alternatives for the treatment of these wounds that contribute to the reduction of pain, time, and epithelialization of the squeal studies are pivotal.^26^ Burn wound healing is an intricate process of inflammation, re-epithelialization, granulation, neovascularization and wound shrinkage.^27^ Several biochemical are involved in burn alleviating process including antioxidants, cytokines and liver and kidney bio-markers.^28^ Antimicrobial, anti-inflammatory, antioxidant, collagen synthesis stimulation, cell proliferative and angiogenic effects are positive activity of phytochemicals that are represented at different stages of burn wound healing.^29^

The skin is the first body defense against penetration of microorganisms and chemical agents.^19^ Thermal injury as a destructive physical and chemical phenomena lead to great problem and death in the world wide.^1^ The wound healing is a spontaneous process and performed by various factors.^12^ There have been a lot of investigation on lesion healing activity of root extracts of some Boraginaceae species, and alkannin, shikonin and their derivatives.^30^ Excellent wound healing properties of Alkannin esters in a clinical study on 72 patients suffering from indolent ulcer on the lower part of the leg were shown due to varicose veins.^29^

Another extract of the Boraginaceae species *Lithospermum erythrorhiozon* root and *Macrotomia euchroma* root have been studied by Ozaki *et al.* regarding the accelerative effect on proliferation of granuloma tissue in rats. They proposed that the accelerative effect on proliferation of granuloma tissue are dependent firstly on the total content of naphthoquinone derivatives while, the accelerative effect convinced by ether extract might be an extra effect of these naphthoquinone derivatives.^31^

In this study, we showed that among different doses of root *O. bulbotrichum* DC extract, the dose of 5% had the best effect for burn improvement. Probably this healing effect is due to presence shikonin as previous studies confirmed that this compound inhibits the biosynthesis of leukotriene B4 and has inflammatory effects.^19^ These compounds are used as new drugs for wound healing nowadays.^15^^,^^19^ In another study, it was shown that the extract (1:1) n- hexane- dichloroethyl Onosma methane and 5,8-o-dimethyl acetyl shikonin led to stimulation and growth of human fibroblasts.^32^

These results showed that increasing the dose does not exceed interacts that are probably due to the cytotoxic effect of quinone in higher concentrations.^33^ It was shown that alkannin and some of its derivatives had effects against many cancer cells, including cancer cells with KB cytotoxic activity.^34^ Also some shikonin derivatives in concentration of 10 mg/kg/day could completely inhibit the tumor growth in mice. Other studies showed that alkannin derivatives after 5, 7, and 10 days had the highest cytotoxic effects that among them, 5-O-methyl-11-O-acetylalkannin was the most effective one.^35^

One of the important factors that develop cytotoxic effects in higher concentrations is quinone because it increases the formation of oxidative stress and free radicals and DNA damages.^36 ^Many studies showed that naphthoquinone derivatives have toxicity at high concentrations revealing an indirect relationship between the concentration and burn healing.^37^ Fibroblasts play an important role in wound healing.^38^ Collagen is one of the most important structural proteins in connective tissue; that this protein is formed mainly in the skin.^39^ Our study showed that the concentration of this protein in the treatment group with cream 5% was significantly different with other groups. The presence of naphthoquinone provides anti-inflammatory effects, that can lead to stimulation of fibroblast growth, and antioxidant activities.^40^

Naphthoquinone derivative, arnebin-1 (b, b-dimethylacrylalkannin), signiﬁcantly accelerates healing of wounds with or without hydrocortisone treatment.^32^ Reduction in the wound width and a gap length compared with controls and promoted cell proliferation, migration and vessel formation results into formation of a thick granulation tissue and re-epithelization of the wounds revealed by Arnebin-1 treatment.^41^ In our present study, *O. bulbotrichum* DC was more effective.

The data of our study suggest that using *O. bulbotrichum* DC for healing of second degree burns is an effective treatment modality, even the exact mechanism underlying these actions is unclear. The possible mechanisms of healing process may be due to the anti-inflammatory effects of *O. bulbotrichum* that can cause stimulation of fibroblast growth, and antioxidant activities.
